# Secrecy Rate Maximization for Movable Antenna-Aided STAR-RIS in Integrated Sensing and Communication Systems

**DOI:** 10.3390/e27121180

**Published:** 2025-11-21

**Authors:** Guanyi Chen, Gang Wang, Jinlong Wang, Donglai Zhao, Chenxu Wang, Tao Jin, Zhiquan Zhou

**Affiliations:** 1 Communication Research Center, Harbin Institute of Technology, Harbin 150001, China; 20b905042@stu.hit.edu.cn (G.C.); gwang51@hit.edu.cn (G.W.); 2 School of Information Science and Engineering, Harbin Institute of Technology, Weihai 264209, China; wangchenxu@hit.edu.cn (C.W.); jintao@hit.edu.cn (T.J.); zhouzq@hit.edu.cn (Z.Z.); 3 Shandong Provincial Key Laboratory of Marine Electronic Information and Intelligent Unmanned Systems, Harbin Institute of Technology, Weihai 264209, China; 4 Key Laboratory of Cross-Domain Synergy and Comprehensive Support for Unmanned Marine Systems, Ministry of Industry and Information Technology, Harbin Institute of Technology, Harbin 150001, China; 5 School of Measurement-Control Technology and Communications Engineering, Harbin University of Science and Technology, Harbin 150080, China; zdl527@hrbust.edu.cn

**Keywords:** integrated sensing and communication (ISAC), movable antenna (MA), simultaneously transmitting and reflecting reconfigurable intelligent surface (STAR-RIS), physical layer security, resource allocation, successive convex approximation (SCA)

## Abstract

Movable antennas (MAs) and simultaneously transmitting and reflecting reconfigurable intelligent surfaces (STAR-RISs) have recently been investigated to enhance integrated sensing and communication (ISAC) systems. However, prior work has not exploited the spatial flexibility of MAs and the extended coverage of STAR-RIS to simultaneously address security issues. In this paper, a novel MA- and STAR-RIS-assisted secure ISAC system is proposed that involves multiple legitimate users and potential eavesdroppers. To ensure fairness, we formulate a minimum secrecy rate maximization problem by jointly optimizing the active beamforming covariance matrices at the base station (BS), the passive transmitting and reflecting beamforming coefficients at the STAR-RIS, and the spatial positions of the MAs. To address the highly nonconvex optimization problem, we propose an efficient iterative algorithm based on the alternating optimization (AO) framework. Specifically, we leverage semidefinite relaxation (SDR) and successive convex approximation (SCA) techniques to solve the active and passive beamforming subproblems, and the SCA method is also applied to tackle the highly intractable MA position optimization subproblem. Numerical results demonstrate that the secure performance of the proposed MA and STAR-RIS-assisted scheme significantly outperforms that of other benchmark schemes, validating the benefits of the proposed algorithm.

## 1. Introduction

The evolution towards sixth-generation (6G) wireless systems is driven by a vision that extends far beyond incremental increases in data rates. 6G wireless systems may introduce a new era of providing multiple services for users, including holographic communications, augmented reality, and the Internet of Everything. These applications require ultra-reliable, low-latency communication and the real-time awareness of the physical environment of the users. The multiple requirements accelerate the development of the integrated sensing and communications (ISAC) as a fundamental 6G function. ISAC may realize improvements in spectral and energy efficiency, as well as reduced hardware and software costs, by jointly designing the communication and sensing functionalities with shared hardware and spectrum resources. The smooth transition to ISAC in the 6G wireless system is a natural need for resource optimization from an engineering perspective to fuse the digital and physical environments. In certain scenarios, such as industrial automation and autonomous driving, the 6G wireless system must act as a sensing and communication center to serve users by enhancing both functionalities.

Despite the huge potential advantage of the deployment of the ISAC systems, engineering problems must be considered. Firstly, the requirements for higher data rates and sensing accuracy push wireless systems to operate in higher frequency bands, where signals may be blocked, leading to performance degradation. Secondly, the multiple functionalities of the ISAC signals may pose a critical security challenge. Signals transmitted to detect a specific target may be intercepted by potential eavesdroppers, posing a threat to data security and personal privacy. This enhances the importance of Physical Layer Security (PLS), which aims to transmit signals securely by integrating the wireless channels, especially for a robust ISAC wireless communications system.

To address these security challenges, several research papers have focused on the beamforming design. A common objective of the research is to maximize the secrecy rate under multiple constraints, such as the quality of service for communication and sensing. Optimization techniques like semidefinite relaxation (SDR) and successive convex approximation (SCA) are often used to handle the non-convexity of the optimization problem [1]. Researchers have integrated multiple access schemes with the proposed secure beamforming ISAC frameworks, such as Non-Orthogonal Multiple Access (NOMA) [2] and Rate-Splitting Multiple Access (RSMA) [3]. The mobile scenarios have been considered in the optimization problems, such as systems with Autonomous Aerial Vehicles (AAVs), where trajectory planning is jointly optimized with beamforming to maximize the average secrecy rate [4]. Moreover, some research papers have focused on sensing metrics like the Cramér–Rao Bound (CRB) with imperfect channel state information (CSI) to ensure the security performance [5]. Recently, Reconfigurable Intelligent Surfaces (RISs) have been introduced into ISAC systems to control the propagation environment and address blockages, thereby enhancing PLS performance by strengthening legitimate links and suppressing eavesdropping channels [6]. Introducing more degrees of freedom and intelligent propagation control is a key step to mitigate the physical blockages and security threats.

Reconfigurable Intelligent Surfaces (RISs) can reflect incident signals to control the propagation environment to combat physical signal blockages. This function is limited in scenarios where users and eavesdroppers are present in both the transmission and reflection areas. The Simultaneously Transmitting and Reflecting RIS (STAR-RIS) is a key breakthrough in this technology, which can provide full-space coverage [7]. This capability is beneficial for system security and flexibility.

Recent studies have demonstrated the superiority of STAR-RIS in PLS performance across different scenarios. The STAR-RIS can significantly improve the secrecy rate in ISAC networks by controlling signals in the full-space [8,9]. Its effectiveness has been confirmed in the following research papers, enhancing security in multi-user NOMA systems [10] and enabling robust transmissions with uncertain eavesdropper locations and imperfect channel state information [11,12]. Furthermore, STAR-RIS can be used for joint secure and covert communications; the full-space control is leveraged to transmit confidential data while hiding the communication itself [13,14,15]. The active STAR-RIS has been studied to further amplify signals to mitigate severe path loss, providing security enhancement against jamming and eavesdropping [16]. The growing interest in related research papers has demonstrated that the full-space control capability of the STAR-RIS fulfills the need for ISAC systems. The communication users, sensing targets, and potential eavesdroppers may be distributed randomly in the full space. The STAR-RIS can provide flexibility and security at the system design level.

Traditional wireless systems have Fixed-Position Antenna (FPA) arrays that remain in static positions. The recently proposed concept of Movable Antennas (MAs) introduces a new degree of freedom by allowing antenna positions to be dynamically and continuously adjusted within a certain region [17]. This movement provides spatial diversity, enabling the wireless communication system to intelligently reconfigure the wireless channel.  An MA-aided system can seek positions that maximize the desired signal and minimize interference, thereby enhancing performance in communication and sensing. This function provides a powerful degree of freedom for system optimization.

The potential benefits of MAs have been studied in different scenarios. There is a growing interest in its application to ISAC and PLS. MAs have been shown to improve the trade-off between sensing and communication performance in the ISAC area. MA-aided ISAC systems can enhance the sensing and communication rate region [18], minimize transmit power [19], and improve beam pattern gains [20] by jointly optimizing antenna positions with transmit and receive beamforming. This improvement has been validated in multiple scenarios, including bistatic systems for airborne vehicles [21], full-duplex monostatic systems with self-interference mitigation [22], and even in the near-field communication scenario [23,24]. The integration of MAs with other technologies is also important. For instance, the integration of MAs with RIS has been shown to further boost ISAC performance in dead zones [25].

Recently, MAs have been extended to enhance the PLS performance of the wireless systems. Several research papers have shown that MAs can increase the secrecy rate by jointly optimizing the beamforming and antenna positions, even with imperfect eavesdropper channel state information [26,27]. This function has been introduced in multiple security-related scenarios, including covert communications [28] and secure simultaneous wireless information and power transfer (SWIPT) [29]. Several works have already combined these promising technologies to investigate secure beamforming design in MA-aided ISAC systems. Another paper has used deep reinforcement learning to handle complex scenarios [30]. These works validate the fact that MAs are an important tool for enhancing the security performance of the 6G wireless system by providing additional spatial degrees of freedom.

While the ISAC, STAR-RIS, and MAs have been investigated individually. The integration of these technologies into a secure framework remains a critical research direction.  The existing papers lack a comprehensive design that jointly leverages the full-space coverage of STAR-RIS and the additional spatial DoFs of MAs to address eavesdropping in a multi-user ISAC system.

The primary motivation for this work is to fill this gap.  However, the joint design presents a huge optimization challenge. The optimization problem needs to optimize the BS beamforming, the passive beamforming at the STAR-RIS, and the position variables of the MAs. This optimization problem is highly non-convex, with multiple constraints, including rank-one constraints on the covariance matrices, unit-modulus constraints on the STAR-RIS coefficients, and non-convex inter-antenna distance constraints for the MAs. This is intractable for the standard convex optimization solvers due to the coupled variables in the optimization problem. There is a need to develop a novel, computationally efficient algorithmic solution to the optimization problem.

This paper demonstrates a secure MA-aided STAR-RIS ISAC system. The main contributions are summarized as follows:

- We propose a novel secure ISAC framework that integrates MAs at the BS with a STAR-RIS. This architecture combines the spatial degree of freedom of MAs with the STAR-RIS, which can enhance the communication security and sensing performance in the meantime.
- We formulate an optimization problem that maximizes the max-min secrecy rate to ensure fairness. This framework optimizes the BS active beamforming, STAR-RIS passive beamforming, and MA positions iteratively. And the optimization problem is subject to constraints on sensing SNR, user QoS, total transmit power, and the physical limitations of the hardware.
- We develop an efficient algorithm based on the Alternating Optimization (AO) framework to solve the highly non-convex optimization problem. We decompose the optimization problem into several subproblems by leveraging SCA and SDR techniques. The decomposition provides a high-quality solution efficiently.
- We provided numerical results that validated the effectiveness of the proposed MA-aided STAR-RIS scheme. The results show visible gains in secrecy rate compared to other benchmark schemes, which demonstrates the benefits of the integrated design.

The remainder of this paper is organized as follows. In Section 2, we present the system model for the movable antenna and STAR-RIS-aided ISAC system. And we formulate the optimization problem for maximizing the minimum secrecy rate. In Section 3, we propose an efficient algorithm based on the alternating optimization (AO) framework to solve the formulated non-convex problem by decomposing it into three subproblems. Section 4 provides extensive simulation results to validate the effectiveness and superiority of our proposed scheme. Finally, Section 5 concludes the paper.

Notations: Matrices and vectors are denoted by bold uppercase and lowercase letters, respectively. ${\left(\cdot \right)}^{T}$, ${\left(\cdot \right)}^{*}$, and ${\left(\cdot \right)}^{H}$ denote the transpose, conjugate, and conjugate transpose, respectively. E[·] denotes the statistical expectation. $C^{M\times N}$ denotes the space of M×N complex matrices. diag(a) denotes a diagonal matrix with the elements of the vector a on its main diagonal. Tr(A) and rank(A) denote the trace and rank of a matrix A, respectively. A⪰0 indicates that A is a positive semidefinite matrix. The description of key symbols is summarized in Table 1.

## 2. System Model and Problem Formulation

### 2.1. System Model

**Figure 1:**
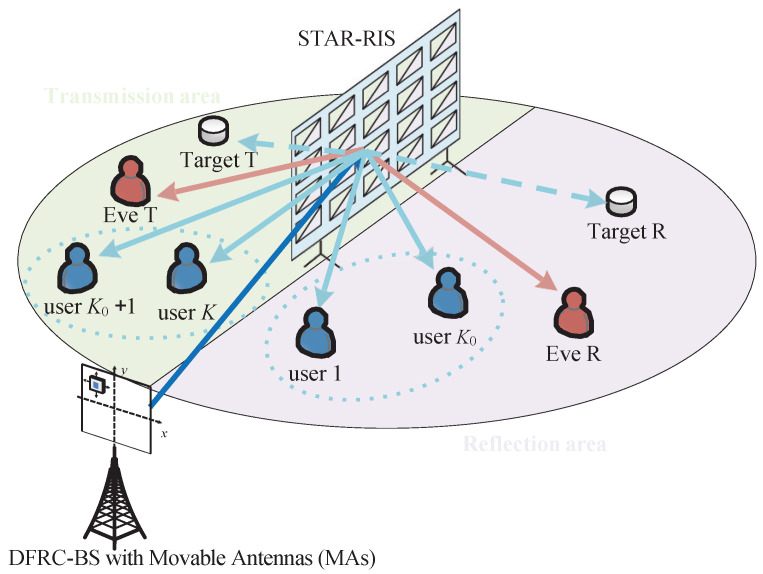
System model for the MA-aided STAR-RIS ISAC system.

As illustrated in Figure 1, we consider a downlink ISAC system where a base station (BS) equipped with *M* movable antennas (MAs) serves a set of *K* legitimate communication users (CUs), denoted by K={1,…,K}, and simultaneously performs sensing tasks. Each CU and the potential eavesdropper (Eve) are assumed to be equipped with a single antenna. The system is threatened by Eve, who attempts to intercept the information transmitted to the CUs. To enhance performance and provide full-space coverage, a STAR-RIS with *N* elements is deployed to assist the BS. In this work, we assume ideal STAR-RIS elements with continuous phase shifts and amplitudes, which provides a performance benchmark. We acknowledge that practical STAR-RIS implementations often employ discrete phase shifts (e.g., 1-bit or 2-bit quantization) to reduce hardware cost and complexity. The performance analysis under such practical hardware constraints, as well as the impact of mutual coupling between antennas, is a relevant topic for future research. As explicitly shown in Figure 1, we assume that a subset of users, $K_{t}$, and a sensing target are located in the transmission space of the STAR-RIS, while the remaining users, $K_{r}$, and Eve are in the reflection space, where $K=K_{t}\cup K_{r}$.

The transmit signal at the BS is a superposition of the communication signals for all users and a dedicated radar signal, given by

(1) $$x=\sum_{k=1}^{K}w_{k}s_{k}+w_{s}s_{s},$$

where $w_{k}\in C^{M\times 1}$ and $s_{k}$ are the beamforming vector and the data symbol for the *k*-th CU, respectively. $w_{s}\in C^{M\times 1}$ is the beamforming vector for the dedicated radar signal $s_{s}$. We assume that the data symbols $s_{k}$ and the sensing symbol $s_{s}$ are statistically independent and drawn from a Gaussian codebook. Without loss of generality, they are normalized to have zero mean and unit variance, i.e., $E\left[s_{j}s_{k}^{*}\right]=\delta_{jk}$ and $E[s_{s}s_{s}^{*}]=1$. It is important to note that $w_{s}s_{s}$ is a dedicated radar signal used for sensing purposes only and is not intended for any of the *K* communication users.

The total transmit covariance matrix at the BS is then given by the following:

(2) $$R_{\mathrm{total}}=E\left[xx^{H}\right]=\sum_{k=1}^{K}w_{k}w_{k}^{H}+w_{s}w_{s}^{H}=\sum_{k=1}^{K}R_{k}+R_{s},$$

where $R_{k}=w_{k}w_{k}^{H}$ and $R_{s}=w_{s}w_{s}^{H}$ are the covariance matrices of the communication signal for user *k* and the sensing signal, respectively. Note that $R_{k}$ and $R_{s}$ are rank-one matrices, which corresponds to the case of single-stream transmission for each communication user and the sensing purpose. The total transmit power at the BS is constrained by $P_{max}$, i.e., $\mathrm{Tr}\left(R_{\mathrm{total}}\right)\leq P_{max}$.

### 2.2. Channel Model

In this work, we adopt a practical far-field channel model that incorporates both line-of-sight (LoS) and non-line-of-sight (NLoS) components. Channels originating from the BS depend on the MA positions, while other channels are modeled as quasi-static.

Let $T=\left[t_{1},\ldots ,t_{M}\right]\in R^{2\times M}$ denote the coordinate matrix of the *M* MAs, where $t_{m}\in C$ is the position of the *m*-th antenna, confined within a continuous two-dimensional region C. Following the field-response model for MAs [17], the channels from the BS are modeled as follows:

- **BS-to-STAR-RIS Channel:** The channel from the BS’s MAs to the STAR-RIS is denoted by $H_{br}\in C^{N\times M}$ and can be expressed as follows:
  (3) $$H_{br}\left(T\right)=A_{r}\Sigma_{br}G_{t}\left(T\right),$$
  where $A_{r}\in C^{N\times L_{br}}$ is the array response matrix at the STAR-RIS; $\Sigma_{br}\in C^{L_{br}\times L_{br}}$ is a diagonal matrix containing the complex gains of the $L_{br}$ paths; and $G_{t}\left(T\right)\in C^{L_{br}\times M}$ is the field response matrix at the BS. The (l,m)-th element of $G_{t}\left(T\right)$ is given by $g_{l,m}\left(t_{m}\right)=e^{j\frac{2\pi }{\lambda }a_{t,l}^{T}t_{m}}$, where λ is the carrier wavelength and $a_{t,l}$ is the direction vector of the *l*-th path from the BS side.
- **BS-to-CU/Eve Direct Channels:** Similarly, the direct channels from the BS to the *k*-th CU and Eve, denoted by $h_{bk}^{H}\left(T\right)\in C^{1\times M}$ and $h_{be}^{H}\left(T\right)\in C^{1\times M}$ respectively, are also functions of the MA positions. Their models are consistent with the field-response framework, e.g., $h_{bk}^{H}\left(T\right)=\sigma_{bk}^{T}G_{t}\left(T\right)$, where $\sigma_{bk}\in C^{L_{bk}\times 1}$ contains the path gains for the BS-to-CU link.

The channels from the STAR-RIS to other nodes are independent of the MA positions and are modeled as Rician fading channels to capture both LoS and NLoS effects. These channels, namely from the STAR-RIS to the *k*-th CU ($h_{rk}^{H}\in C^{1\times N}$), to Eve ($h_{re}^{H}\in C^{1\times N}$), and to the sensing target ($h_{rt}^{H}\in C^{1\times N}$), are assumed to be known through channel estimation.

The STAR-RIS manipulates the incident signal using the energy splitting (ES) protocol. The transmission and reflection actions are characterized by diagonal coefficient matrices $\Theta_{t}=\mathrm{diag}\left(v_{t}\right)$ and $\Theta_{r}=\mathrm{diag}\left(v_{r}\right)$, respectively. Here, the beamforming vectors are defined as $v_{t}={\left[\sqrt{\beta_{1}^{t}}e^{j\theta_{1}},\ldots ,\sqrt{\beta_{N}^{t}}e^{j\theta_{N}}\right]}^{T}$ and $v_{r}={\left[\sqrt{\beta_{1}^{r}}e^{j\theta_{1}},\ldots ,\sqrt{\beta_{N}^{r}}e^{j\theta_{N}}\right]}^{T}$. For each element n∈{1,…,N}, $\beta_{n}^{t},\beta_{n}^{r}\in \left[0,1\right]$ are the energy splitting ratios for transmission and reflection, which satisfy the energy conservation law $\beta_{n}^{t}+\beta_{n}^{r}=1$. Furthermore, $\theta_{n}\in \left[0,2\pi \right)$ represents the common phase shift applied to both transmitted and reflected signals.

We assume that the CSI of all channels is perfectly known at the BS. This ideal assumption is common for enabling tractable analysis and establishing performance upper bounds. Acquiring CSI in practice would require sophisticated channel estimation techniques, especially for the MA-dependent channels. The design of robust algorithms under imperfect CSI is a significant challenge for future research investigation.

### 2.3. Communication and Sensing Metrics

In this section, we define the performance metrics for both communication and sensing based on the received signals at the CUs, Eve, and the BS.

#### 2.3.1. Communication Metrics

First, we formulate the effective channel from the BS to any CU *k*. Depending on its location, the effective channel is a combination of the direct BS-CU link and the cascaded BS-STAR-RIS-CU link. For a CU $k\in K_{t}$ (in the transmission space), the effective channel is given by the following:

(4) $$h_{k}^{H}\left(T,v_{t}\right)=h_{rk}^{H}\Theta_{t}H_{br}\left(T\right)+h_{bk}^{H}\left(T\right).$$

For a CU $k\in K_{r}$ (in the reflection space), the transmission matrix $\Theta_{t}$ is simply replaced by the reflection matrix $\Theta_{r}$.

The received signal at CU *k* can thus be written as follows:

(5) $$y_{k}=h_{k}^{H}\left(w_{k}s_{k}+\sum_{i\neq k}^{K}w_{i}s_{i}+w_{s}s_{s}\right)+n_{k},$$

where $n_{k}∼\mathrm{CN}\left(0,\sigma_{k}^{2}\right)$ is the additive white Gaussian noise (AWGN) at the receiver of CU *k*. Based on this, the signal-to-interference-plus-noise ratio (SINR) at CU *k* is formulated as follows:

(6) $$\gamma_{k}=\frac{\mathrm{Tr}(R_{k}H_{k})}{\sum_{i\neq k}\mathrm{Tr}\left(R_{i}H_{k}\right)+\mathrm{Tr}\left(R_{s}H_{k}\right)+\sigma_{k}^{2}},$$

where $H_{k}=h_{k}h_{k}^{H}$ is the effective channel covariance matrix for user *k*. The numerator represents the desired signal power, while the denominator comprises the inter-user interference, interference from the sensing signal, and the noise power.

Similarly, the effective channel from the BS to Eve, located in the reflection space, is as follows:

(7) $$h_{e}^{H}\left(T,v_{r}\right)=h_{re}^{H}\Theta_{r}H_{br}\left(T\right)+h_{be}^{H}\left(T\right).$$

The SINR at Eve when attempting to decode the signal of user *k* is then as follows:

(8) $$\gamma_{e,k}=\frac{\mathrm{Tr}(R_{k}H_{e})}{\sum_{i\neq k}\mathrm{Tr}\left(R_{i}H_{e}\right)+\mathrm{Tr}\left(R_{s}H_{e}\right)+\sigma_{e}^{2}},$$

where $H_{e}=h_{e}h_{e}^{H}$ and $\sigma_{e}^{2}$ is the noise power at Eve. This model assumes the eavesdropper attempts to decode the signal for user *k* while treating all other signals (for i≠k and the sensing signal) as noise, without the capability for multi-user detection or successive interference cancellation.

The achievable secrecy rate for user *k*, which quantifies the secure transmission performance against eavesdropping, is defined as the difference between the achievable rate at the CU and that at Eve:

(9) $$R_{sec,k}={\left[\log_{2}\left(1+\gamma_{k}\right)-\log_{2}\left(1+\gamma_{e,k}\right)\right]}^{+},$$

where ${\left[\cdot \right]}^{+}≜\max\left(0,\cdot \right)$ ensures the non-negativity of the rate.

#### 2.3.2. Sensing Metric

For the sensing task, the BS analyzes the echo of the entire transmitted signal x. We assume a joint-waveform ISAC model where the BS, as the sensing receiver, has perfect knowledge of the transmitted communication and sensing symbols ($s_{k}$ and $s_{s}$). Therefore, the echoes generated by the communication signals are not treated as interference; rather, they are coherently processed with the echoes from the sensing signal to enhance the sensing performance. The total received echo signal power is thus proportional to the power of the entire transmitted signal $R_{\mathrm{total}}$. This leads to the definition of the sensing SNR in Equation (10). For sensing, the BS transmits signals and analyzes the echoes reflected from the target. The round-trip sensing channel is the cascaded BS-RIS-Target-RIS-BS link, whose channel vector is given by $h_{s}^{H}=\alpha h_{rt}^{H}\Theta_{t}H_{br}\left(T\right)$, where α accounts for the radar cross-section (RCS) and path loss of the target link. For simplicity, we absorb this scalar into the channel definition. The received echo signal at the BS is processed to estimate the target’s parameters. The signal-to-noise ratio (SNR) for sensing is a key performance metric, given by the following:

(10) $$\gamma_{s}=\frac{E[|h_{s}^{H}x{|}^{2}]}{\sigma_{s}^{2}}=\frac{\mathrm{Tr}(R_{\mathrm{total}}H_{s})}{\sigma_{s}^{2}},$$

where $H_{s}=h_{s}h_{s}^{H}$ is the sensing channel covariance matrix, and $\sigma_{s}^{2}$ is the noise power at the BS’s sensing receiver.

We adopt the sensing SNR as the performance metric, as it is widely used in related research papers and directly reflects the strength of the received echo signal. More specific metrics, such as the Cramér–Rao Bound (CRB) or detection probability, could also be used. As this paper focuses on the integration of MAs and STAR-RIS, we utilize the SNR metric as a fundamental and tractable measure of sensing performance. And the investigation of CRB-based optimization as a direction for future work.

### 2.4. Problem Formulation

Our goal is to maximize the minimum secrecy rate among all CUs by jointly optimizing the BS active beamforming covariance matrices $\left\{R_{k},R_{s}\right\}$, the STAR-RIS passive beamforming vectors $\left\{v_{t},v_{r}\right\}$, and the MA positions T. The max-min fairness problem is formulated as follows:

(11a) $$\begin{array}{cc} \max_{\begin{array}{c} \left\{R_{k}\right\},R_{s},v_{t},\\ v_{r},T \end{array}}\; & \min_{k\in K}\;R_{sec,k} \end{array}$$

(11b) $$\begin{array}{cc} s.t.\; & \gamma_{s}\geq \Gamma_{s}, \end{array}$$

(11c) $$\begin{array}{cc} \; & \gamma_{k}\geq \Gamma_{k},\;\forall k\in K, \end{array}$$

(11d) $$\begin{array}{cc} \; & \mathrm{Tr}\left(\sum_{k=1}^{K}R_{k}+R_{s}\right)\leq P_{max}, \end{array}$$

(11e) $$\begin{array}{cc} \; & R_{k}⪰0,R_{s}⪰0,\;\forall k\in K, \end{array}$$

(11f) $$\begin{array}{cc} \; & \mathrm{rank}\left(R_{k}\right)=1,\mathrm{rank}\left(R_{s}\right)=1,\;\forall k\in K, \end{array}$$

(11g) $$\begin{array}{cc} \; & |{\left[v_{t}\right]}_{n}{|}^{2}+{\left|{\left[v_{r}\right]}_{n}\right|}^{2}=1,\;\forall n=1,\ldots ,N, \end{array}$$

(11h) $$\begin{array}{cc} \; & ∠\left({\left[v_{t}\right]}_{n}\right)=∠\left({\left[v_{r}\right]}_{n}\right),\;\forall n=1,\ldots ,N, \end{array}$$

(11i) $$\begin{array}{cc} \; & \left|\right|t_{m}-t_{j}{\left|\right|}_{2}\geq D_{min},\;\forall m\neq j, \end{array}$$

(11j) $$\begin{array}{cc} \; & t_{m}\in C,\;\forall m=1,\ldots ,M. \end{array}$$

Here, the objective function (11a) aims to maximize the minimum secrecy rate to ensure fairness. The objective function (11a) is fully defined by Equation (9), which in turn depends on the SINR definitions in Equations (6) and (8). The constraints are detailed as follows:

- (11b) is the sensing SNR constraint, where $\Gamma_{s}$ is the minimum required SNR at the BS to guarantee the sensing performance.
- (11c) ensures the quality of service (QoS) for communication, where $\Gamma_{k}$ is the minimum required SINR for user *k*.
- (11d) is the total transmit power constraint at the BS. And $P_{max}$ is the maximum power budget.
- (11e) ensures that the covariance matrices are positive semidefinite.
- (11f) is the rank-one constraint.
- (11g) is the energy conservation constraint for each element of the STAR-RIS.
- (11h) is the phase consistency constraint.
- (11i) is the minimum distance constraint for the MAs. And $D_{min}$ is the minimum separation to avoid the mutual coupling effect.
- (11j) defines the position of each MA, $t_{m}$, to a continuous region C.

Problem (11) is highly non-convex due to the non-convex max-min objective function, the coupled optimization variables in the SINR and SNR expressions, the non-convex rank-one constraints (11f), and the non-convex constraints related to the STAR-RIS phases (11h) and MA positions (11i).

## 3. Proposed Solution

The formulated problem (11) is highly intractable. We propose an efficient iterative algorithm based on the alternating optimization (AO) framework. The core idea is to divide the optimization problem into three sub-problems—active beamforming matrices $\left\{R_{k},R_{s}\right\}$, passive beamforming vectors $\left\{v_{t},v_{r}\right\}$, and MA positions T. Then optimize one problem at a time. This process is repeated until convergence. The overall AO algorithm consists of three main blocks that are solved iteratively. Each block (Algorithms 1–3) is solved using an SCA-based method, which involves solving a convex program at each step. This ensures that the objective of each subproblem is non-decreasing. As each step of the AO framework improves (or at least does not degrade) the overall objective function, the proposed Algorithm 4 is guaranteed to converge monotonically to a locally optimal solution.

### 3.1. Active Beamforming Optimization

With fixed passive beamforming and MA positions, we first apply SDR by dropping the rank-one constraints (11f). The subproblem for optimizing the active beamforming covariance matrices, denoted by $R={\left\{R_{k}\right\}}_{k=1}^{K}\cup \left\{R_{s}\right\}$, is as follows:

(12a) $$\begin{array}{cc} \max_{R}\; & \min_{k\in K}\;R_{sec,k}\left(R\right) \end{array}$$

(12b) $$\begin{array}{cc} s.t.\; & (11b),(11c),(11d),(11e). \end{array}$$

This problem is still non-convex due to the max-min objective function. We can rewrite it by introducing an epigraph variable τ as follows:

(13a) $$\begin{array}{cc} \max_{R,\tau }\; & \tau \end{array}$$

(13b) $$\begin{array}{cc} s.t.\; & R_{sec,k}\left(R\right)\geq \tau ,\;\forall k\in K, \end{array}$$

(13c) $$\begin{array}{cc} \; & \mathrm{Constraints}\;(11b),(11c),(11d),(11e). \end{array}$$

The main challenges are the non-concave secrecy rate constraint (13b) and the rank-one constraint. We propose an iterative algorithm based on the SCA framework to deal with this.

We approximate the function $R_{sec,k}\left(R\right)$ with a concave lower bound at each iteration of the SCA algorithm. Then let $R^{\left(i\right)}$ be the set of covariance matrices obtained at the *i*-the SCA iteration. For the (i+1)-th iteration, the constraint (13b) is replaced by its first-order Taylor expansion:

(14) $$R_{sec,k}\left(R^{\left(i\right)}\right)+\mathrm{Tr}\left(\nabla_{R}R_{sec,k}{\left(R^{\left(i\right)}\right)}^{T}\left(R-R^{\left(i\right)}\right)\right)\geq \tau .$$

The gradient $\nabla_{R}R_{sec,k}\left(R^{\left(i\right)}\right)$ can be derived. This transforms the constraint into a linear constraint.

Second, simply dropping the rank-one constraint often yields a solution with a rank greater than one. To address this, we employ an iteratively reweighted penalty method to encourage rank-one solutions. This method is based on the property that a positive semidefinite matrix R is rank-one if and only if $\mathrm{Tr}\left(R\right)-\lambda_{\max}\left(R\right)=0$, where $\lambda_{\max}\left(R\right)$ is the maximum eigenvalue of R. We add a penalty term to the objective function to minimize this difference. Since $\lambda_{\max}\left(R\right)$ is a convex function, we approximate it with its affine lower bound obtained from the first-order Taylor expansion at $R^{\left(i\right)}$:

(15) $$\lambda_{\max}\left(R\right)\geq \lambda_{\max}\left(R^{\left(i\right)}\right)+\mathrm{Tr}\left({\left(v^{\left(i\right)}{\left(v^{\left(i\right)}\right)}^{H}\right)}^{T}\left(R-R^{\left(i\right)}\right)\right),$$

where $v^{\left(i\right)}$ is the eigenvector corresponding to the maximum eigenvalue of $R^{\left(i\right)}$. Let us denote the right-hand side of the inequality as ${\hat{\lambda }}_{\max}\left(R;R^{\left(i\right)}\right)$.

By combining the SCA approximation for the secrecy rate and the penalty method for the rank-one constraint, the active beamforming optimization subproblem at each iteration is formulated as the following standard SDP:

(16a) $$\begin{array}{cc} \max_{R,\tau }\; & \tau -\sum_{k=1}^{K}\rho_{k}\left(\mathrm{Tr}\left(R_{k}\right)-{\hat{\lambda }}_{\max}\left(R_{k};R_{k}^{\left(i\right)}\right)\right)-\rho_{s}\left(\mathrm{Tr}\left(R_{s}\right)-{\hat{\lambda }}_{\max}\left(R_{s};R_{s}^{\left(i\right)}\right)\right) \end{array}$$

(16b) $$\begin{array}{cc} s.t.\; & R_{sec,k}\left(R^{\left(i\right)}\right)+\sum_{R_{j}\in R}\mathrm{Tr}\left({\left(\nabla_{R_{j}}R_{sec,k}\left(R^{\left(i\right)}\right)\right)}^{T}\left(R_{j}-R_{j}^{\left(i\right)}\right)\right)\geq \tau ,\;\forall k\in K, \end{array}$$

(16c) $$\begin{array}{cc} \; & \mathrm{Tr}\left(R_{k}H_{k}\right)\geq \Gamma_{k}\left(\sum_{j\neq k}\mathrm{Tr}\left(R_{j}H_{k}\right)+\mathrm{Tr}\left(R_{s}H_{k}\right)+\sigma_{k}^{2}\right),\;\forall k\in K, \end{array}$$

(16d) $$\begin{array}{cc} \; & \mathrm{Tr}\left(\left(\sum_{k=1}^{K}R_{k}+R_{s}\right)H_{s}\right)\geq \Gamma_{s}\sigma_{s}^{2}, \end{array}$$

(16e) $$\begin{array}{cc} \; & (11d),(11e). \end{array}$$

Here, $\rho_{k}>0$ and $\rho_{s}>0$ are penalty factors that are gradually increased in each iteration to progressively enforce the rank-one property. This SDP can be efficiently solved using standard convex optimization solvers like CVX. If the resulting matrices are not exactly rank-one, a rank-one solution can be constructed from the dominant eigenvector, which is a standard procedure for this method.

The overall iterative procedure for solving the active beamforming subproblem (12) is summarized in Algorithm 1. In each iteration, we solve the convex SDP problem (16) to obtain an updated set of covariance matrices. The penalty factors are progressively increased to ensure that the resulting solution converges to a rank-one solution. After the algorithm converges, a standard rank-one approximation step is performed if the resulting matrices are not of perfect rank one.
**Algorithm 1** SCA-based Algorithm for Active Beamforming Optimization  1:*Input:* Fixed passive beamforming $\left\{v_{t},v_{r}\right\}$ and MA positions T.  2:*Initialize:* Feasible covariance matrices $R^{\left(0\right)}=\left\{{\left\{R_{k}^{\left(0\right)}\right\}}_{k=1}^{K},R_{s}^{\left(0\right)}\right\}$, SCA iteration counter i=0, penalty factors $\rho_{k}^{\left(0\right)},\rho_{s}^{\left(0\right)}$, and update parameter β>1.  3:**repeat**  4:   Obtain the concave lower bound of $R_{sec,k}$ for all k∈K by applying the first-order Taylor expansion at $R^{\left(i\right)}$.  5:   Find the principal eigenvectors $v_{k}^{\left(i\right)}$ of $R_{k}^{\left(i\right)}$ and $v_{s}^{\left(i\right)}$ of $R_{s}^{\left(i\right)}$ to construct the affine lower bound of the maximum eigenvalues ${\hat{\lambda }}_{\max}\left(R_{k};R_{k}^{\left(i\right)}\right)$ and ${\hat{\lambda }}_{\max}\left(R_{s};R_{s}^{\left(i\right)}\right)$.  6:   Solve the convex SDP subproblem in (16) to obtain the solution $R^{*}$.  7:   Update the covariance matrices: $R^{\left(i+1\right)}\leftarrow R^{*}$.  8:   Update penalty factors: $\rho_{k}^{\left(i+1\right)}\leftarrow \beta \rho_{k}^{\left(i\right)}$, $\rho_{s}^{\left(i+1\right)}\leftarrow \beta \rho_{s}^{\left(i\right)}$.  9:   i←i+1.10:**until** the objective function converges or the maximum number of iterations is reached.11:*Output:* The optimized rank-one covariance matrices $\left\{R_{k}^{*},R_{s}^{*}\right\}$, obtained by extracting the principal eigenvectors from the final matrices in $R^{\left(i\right)}$.

### 3.2. Passive Beamforming Optimization

In this subsection, with the active beamforming matrices $\left\{F_{k},F_{s}\right\}$ and MA positions P fixed from the previous steps, we focus on optimizing the passive beamforming at the STAR-RIS. This involves finding the optimal reflection and transmission coefficient matrices, denoted by $\Phi_{r}$ and $\Phi_{t}$. The optimization subproblem can be formulated as follows:

(17a) $$\begin{array}{cc} \left(P3\right):\max_{v_{r},v_{t},q_{3}}\; & q_{3} \end{array}$$

(17b) $$\begin{array}{cc} s.t.\; & R_{s,k}^{\sec}\geq q_{3},\;\forall k\in K, \end{array}$$

(17c) $$\begin{array}{cc} \; & P_{\mathrm{sen},m}\geq p_{\mathrm{th}},\;m\in \left\{r,t\right\}, \end{array}$$

(17d) $$\begin{array}{cc} \; & |{\left[v_{r}\right]}_{n}{|}^{2}+{\left|{\left[v_{t}\right]}_{n}\right|}^{2}=1,\;\forall n\in \left\{1,\ldots ,N\right\}, \end{array}$$

(17e) $$\begin{array}{cc} \; & \mathrm{rank}\left(V_{r}\right)=1,\;\mathrm{rank}\left(V_{t}\right)=1, \end{array}$$

where we have defined $V_{r}=v_{r}v_{r}^{H}$ and $V_{t}=v_{t}v_{t}^{H}$. This problem is non-convex due to the coupled variables in the secrecy rate expression (17b) and the non-convex unit-modulus and rank-one constraints in (17d) and (17e). To tackle this, we employ a combination of Semidefinite Relaxation (SDR) and Successive Convex Approximation (SCA), augmented by a penalty method to handle the rank-one constraint.

First, we apply SDR by dropping the non-convex rank-one constraints (17e), which relaxes $V_{r}$ and $V_{t}$ to be positive semidefinite matrices, i.e., $V_{r}⪰0$ and $V_{t}⪰0$. The unit-modulus constraint (17d) is equivalent to constraining the diagonal elements of the sum of these matrices, i.e., $\mathrm{diag}\left(V_{r}\right)+\mathrm{diag}\left(V_{t}\right)=1_{N}$.

The secrecy rate constraint (17b) remains non-convex. We can rewrite the achievable rates for user *k* and the eavesdropper as functions of $V_{r}$ and $V_{t}$. For instance, for a transmission user $k\in K_{t}$:

(18) $$\begin{array}{cc} R_{u,k} & =\log_{2}\left(1+\mathrm{Tr}\left(U_{u,k}^{t}V_{t}\right)\right), \end{array}$$

(19) $$\begin{array}{cc} R_{e,k} & =\log_{2}\left(1+\mathrm{Tr}\left(S_{e,k}^{t}V_{t}\right)+\mathrm{Tr}\left(S_{e,k}^{r}V_{r}\right)\right), \end{array}$$

where the matrices $U_{u,k}^{t}$, $S_{e,k}^{t}$, and $S_{e,k}^{r}$ are derived from the channel matrices and active beamformers. Similar expressions can be obtained for reflection users. The non-convexity arises from the difference of two concave functions. We apply SCA by computing the first-order Taylor expansion of the concave parts of the rate expressions. For example, for user *k*, we obtain the convex lower bound ${\tilde{R}}_{u,k}$ for $R_{u,k}$ and a convex upper bound ${\tilde{R}}_{e,k}$ for $R_{e,k}$ around the feasible points $V_{r}^{\left(i\right)}$ and $V_{t}^{\left(i\right)}$ from the *i*-th iteration.

Even after applying SDR and SCA, the rank-one constraints are still violated. To enforce the rank-one property, we introduce a penalty term into the objective function. The rank of a semidefinite matrix V is one if and only if $\mathrm{Tr}\left(V\right)-\lambda_{\max}\left(V\right)=0$, where $\lambda_{\max}\left(\cdot \right)$ is the maximum eigenvalue. Since $\lambda_{\max}\left(V\right)$ is a convex but non-smooth function, we approximate it by its upper bound $u^{H}Vu$, where u is the eigenvector corresponding to the maximum eigenvalue of V in the previous iteration. By linearizing this approximation, we construct a penalty term that is concave with respect to V. This leads to the penalized objective function for the (i+1)-th SCA iteration:

(20) $$\begin{array}{c} \max_{V_{r},V_{t},q_{3}}\;q_{3}-\rho_{r}^{\left(i\right)}\left(\mathrm{Tr}\left(V_{r}\right)-\Psi_{r}^{\left(i\right)}\right)-\rho_{t}^{\left(i\right)}\left(\mathrm{Tr}\left(V_{t}\right)-\Psi_{t}^{\left(i\right)}\right), \end{array}$$

where $\rho_{r}^{\left(i\right)}$ and $\rho_{t}^{\left(i\right)}$ are penalty factors that are gradually increased with each iteration (i.e., $\rho^{\left(i+1\right)}=\beta \rho^{\left(i\right)}$ with β>1). The terms $\Psi_{r}^{\left(i\right)}$ and $\Psi_{t}^{\left(i\right)}$ are the first-order approximations of the maximum eigenvalues of $V_{r}$ and $V_{t}$ at the current iterate, given by the following:

(21) Ψr(i)=(μr(i))HVr(i)μr(i)+2Re{(μr(i))H(Vr−Vr(i))μr(i)},

(22) Ψt(i)=(μt(i))HVt(i)μt(i)+2Re{(μt(i))H(Vt−Vt(i))μt(i)},

where $\mu_{r}^{\left(i\right)}$ and $\mu_{t}^{\left(i\right)}$ are the principal eigenvectors of $V_{r}^{\left(i\right)}$ and $V_{t}^{\left(i\right)}$, respectively.

By integrating the SCA approximations and the penalty terms, the problem at each iteration becomes a standard semidefinite program (SDP), which can be efficiently solved using convex optimization tools like CVX.

Once the algorithm converges, if the resulting matrices $V_{r}^{*}$ and $V_{t}^{*}$ are not perfectly rank-one, we use Gaussian randomization or simply extract the principal eigenvector to construct a high-quality rank-one approximate solution. We apply the eigenvector extraction method: $v^{*}=\sqrt{\lambda_{\max}\left(V^{*}\right)}u_{\max}$, where $u_{\max}$ is the corresponding eigenvector.

The overall procedure for solving the passive beamforming subproblem (P3) is summarized in Algorithm 2. We employ an iterative SCA-based approach, where in each step, a convex SDP is solved. The penalty terms are progressively increased to ensure the eventual recovery of rank-one solutions.
**Algorithm 2** SCA-based Algorithm for Passive Beamforming Optimization  1:*Input:* Fixed active beamforming $\left\{R_{k},R_{s}\right\}$ and MA positions T.  2:*Initialize:* Feasible matrices $V_{r}^{\left(0\right)},V_{t}^{\left(0\right)}$, SCA iteration counter i=0, penalty factors $\rho_{r}^{\left(0\right)},\rho_{t}^{\left(0\right)}$, and update parameter β>1.  3:**repeat**  4:   Compute the first-order Taylor approximations for the secrecy rate constraints at the current point $\left\{V_{r}^{\left(i\right)},V_{t}^{\left(i\right)}\right\}$.  5:   Find the principal eigenvectors of $V_{r}^{\left(i\right)}$ and $V_{t}^{\left(i\right)}$ to update the penalty terms for promoting rank-one solutions.  6:   Solve the convex SDP subproblem formulated in Section 3.2 to obtain the solution $\left\{V_{r}^{*},V_{t}^{*}\right\}$.  7:   Update the matrices: $V_{r}^{\left(i+1\right)}\leftarrow V_{r}^{*}$, $V_{t}^{\left(i+1\right)}\leftarrow V_{t}^{*}$.  8:   Update penalty factors: $\rho_{r}^{\left(i+1\right)}\leftarrow \beta \rho_{r}^{\left(i\right)}$, $\rho_{t}^{\left(i+1\right)}\leftarrow \beta \rho_{t}^{\left(i\right)}$.  9:   i←i+1.10:**until** the objective function converges or the maximum number of iterations is reached.11:*Output:* The optimized rank-one vectors $\left\{v_{r}^{*},v_{t}^{*}\right\}$, recovered from the principal eigenvectors of the final matrices.

### 3.3. MA Position Optimization

With the active and passive beamforming matrices fixed, the subproblem of optimizing the MA positions T remains. This is the most challenging part due to the highly non-linear and non-convex dependency of the channel matrices on T via complex exponential functions. The subproblem can be formulated as follows:

(23a) $$\begin{array}{cc} \max_{T}\; & \min_{k\in K}\;R_{sec,k}\left(T\right) \end{array}$$

(23b) $$\begin{array}{cc} s.t.\; & \gamma_{s}\left(T\right)\geq \Gamma_{s},\;\gamma_{k}\left(T\right)\geq \Gamma_{k},\;\forall k\in K, \end{array}$$

(23c) $$\begin{array}{cc} \; & (11i),(11j). \end{array}$$

Directly solving problem (23) with respect to the entire matrix T is computationally prohibitive.

Instead, we propose an efficient algorithm that combines the block coordinate descent (BCD) and successive convex approximation (SCA) techniques. Specifically, we optimize the position of one MA at a time, while keeping the positions of the other MAs fixed. This process is repeated for all MAs until convergence is achieved.

Let’s focus on optimizing the position of the *m*-th MA, $t_{m}$, with the positions of other antennas $T_{\bar{m}}={\left\{t_{j}\right\}}_{j\neq m}$ being fixed. Let $T^{\left(i\right)}$ be the MA positions obtained at the *i*-th iteration. The subproblem for $t_{m}$ is then solved using the SCA method by replacing the non-convex objective and constraints with their first-order Taylor approximations around the current point $t_{m}^{\left(i\right)}$.

#### 3.3.1. Approximation of Objective and Constraints

By introducing an epigraph variable τ, the objective becomes maximizing τ subject to $R_{sec,k}\left(T\right)\geq \tau$ for all k∈K. We linearize the secrecy rate function at $T^{\left(i\right)}$:

(24) $$R_{sec,k}\left(T^{\left(i\right)}\right)+\nabla_{t_{m}}R_{sec,k}{\left(T^{\left(i\right)}\right)}^{T}\left(t_{m}-t_{m}^{\left(i\right)}\right)\geq \tau .$$

The gradient $\nabla_{t_{m}}R_{sec,k}$ can be computed using the chain rule, as detailed in Section 3.3.1 of the original paper. The key lies in deriving the gradient of the channel matrix $H_{br}\left(T\right)$ with respect to $t_{m}$.

Similarly, the non-convex SINR/SNR constraints are replaced by their linear approximations. For instance, the QoS constraint $\gamma_{k}\left(T\right)\geq \Gamma_{k}$ is rewritten as $f_{k}\left(T\right)\geq 0$ and then approximated as follows:

(25) $$f_{k}\left(T^{\left(i\right)}\right)+\nabla_{t_{m}}f_{k}{\left(T^{\left(i\right)}\right)}^{T}\left(t_{m}-t_{m}^{\left(i\right)}\right)\geq 0.$$

The gradient $\nabla_{t_{m}}f_{k}$ can be derived straightforwardly. The sensing constraint $\gamma_{s}\left(T\right)\geq \Gamma_{s}$ is handled in the same manner.

The inter-antenna distance constraint (11i), $\left|\right|t_{m}-t_{j}{\left|\right|}_{2}\geq D_{min}$ for j≠m, is non-convex as it lower-bounds a convex function. We can obtain a convex inner approximation by applying the first-order Taylor expansion to the convex function $g_{mj}\left(t_{m}\right)=\left|\right|t_{m}-t_{j}{\left|\right|}_{2}$ at the point $t_{m}^{\left(i\right)}$:

(26) $$\left|\right|t_{m}^{\left(i\right)}-t_{j}{\left|\right|}_{2}+\frac{{\left(t_{m}^{\left(i\right)}-t_{j}\right)}^{T}}{∥t_{m}^{\left(i\right)}-t_{j}{∥}_{2}}\left(t_{m}-t_{m}^{\left(i\right)}\right)\geq D_{min},\;\forall j\neq m.$$

This formulation provides a linear and thus convex constraint for the optimization problem.

#### 3.3.2. The Convex Subproblem with Trust Region

By applying the SCA steps, we can formulate a convex program.

However, the first-order approximations are accurate only within a small neighborhood of the expansion point $T^{\left(i\right)}$. To ensure the validity of the approximations and improve the convergence stability of the SCA algorithm, we introduce a trust region constraint that limits the maximum displacement of the MA in each iteration:

(27) $$\left|\right|t_{m}-t_{m}^{\left(i\right)}{\left|\right|}_{2}\leq \Delta_{t},$$

where $\Delta_{t}$ is the trust region radius, which serves as a step-size control parameter.

Finally, by integrating the BCD framework with the SCA method and the trust region constraint, the subproblem to find the updated position $t_{m}^{\left(i+1\right)}$ becomes the following convex program, which can be solved efficiently:

(28a) $$\begin{array}{cc} \max_{t_{m},\tau }\; & \tau \end{array}$$

(28b) $$\begin{array}{cc} s.t.\; & R_{sec,k}\left(T^{\left(i\right)}\right)+\nabla_{t_{m}}R_{sec,k}{\left(T^{\left(i\right)}\right)}^{T}\left(t_{m}-t_{m}^{\left(i\right)}\right)\geq \tau ,\;\forall k\in K, \end{array}$$

(28c) $$\begin{array}{cc} \; & f_{k}\left(T^{\left(i\right)}\right)+\nabla_{t_{m}}f_{k}{\left(T^{\left(i\right)}\right)}^{T}\left(t_{m}-t_{m}^{\left(i\right)}\right)\geq 0,\;\forall k\in K, \end{array}$$

(28d) $$\begin{array}{cc} \; & \mathrm{Linearized}\;\mathrm{sensing}\;\mathrm{constraint}\;\mathrm{for}\;\gamma_{s}\left(T\right)\geq \Gamma_{s}, \end{array}$$

(28e) $$\begin{array}{cc} \; & \left|\right|t_{m}^{\left(i\right)}-t_{j}{\left|\right|}_{2}+\frac{{\left(t_{m}^{\left(i\right)}-t_{j}\right)}^{T}}{∥t_{m}^{\left(i\right)}-t_{j}{∥}_{2}}\left(t_{m}-t_{m}^{\left(i\right)}\right)\geq D_{min},\;\forall j\neq m, \end{array}$$

(28f) $$\begin{array}{cc} \; & \left|\right|t_{m}-t_{m}^{\left(i\right)}{\left|\right|}_{2}\leq \Delta_{t}, \end{array}$$

(28g) $$\begin{array}{cc} \; & t_{m}\in C. \end{array}$$

The overall procedure for solving the passive beamforming subproblem (P3) is summarized in Algorithm 3. We employ an iterative SCA-based approach, where in each step, a convex SDP is solved. The penalty terms are progressively increased to ensure the eventual recovery of rank-one solutions.
**Algorithm 3** BCD-SCA Algorithm for MA Position Optimization  1:*Input:* Fixed active beamforming $\left\{R_{k},R_{s}\right\}$, passive beamforming $\left\{v_{t},v_{r}\right\}$.  2:*Initialize:* MA positions $T^{\left(0\right)}$, BCD iteration counter i=0.  3:**while** objective function has not converged **do**  4:    $T_{\mathrm{temp}}\leftarrow T^{\left(i\right)}$.  5:    **for** m=1,…,M **do**  6:        Initialize $t_{m}^{\left(0\right)}\leftarrow {\left[T_{\mathrm{temp}}\right]}_{m}$ and SCA counter j=0.  7:        **repeat**  8:            Solve the convex subproblem in (28) at point $t_{m}^{\left(j\right)}$ to obtain $t_{m}^{\left(j+1\right)}$.  9:            j←j+1.10:        **until** $\left|\right|t_{m}^{\left(j\right)}-t_{m}^{\left(j-1\right)}\left|\right|<\epsilon_{\mathrm{inner}}$.11:        ${\left[T_{\mathrm{temp}}\right]}_{m}\leftarrow t_{m}^{\left(j\right)}$.12:    **end for**13:    $T^{\left(i+1\right)}\leftarrow T_{\mathrm{temp}}$.14:    i←i+1.15:**end while**16:*Output:* The optimized MA positions $T^{*}\leftarrow T^{\left(i\right)}$.

The overall procedure for solving the problem (11) is summarized in Algorithm 4. We employ an AO framework that iteratively solves three subproblems: active beamforming optimization, passive beamforming optimization, and MA position optimization, until the objective function value converges.
**Algorithm 4** Proposed AO-based Algorithm for Problem (11)  1:*Initialize:* Feasible active beamforming matrices $\left\{R_{k}^{\left(0\right)},R_{s}^{\left(0\right)}\right\}$, passive beamforming vectors $\left\{v_{t}^{\left(0\right)},v_{r}^{\left(0\right)}\right\}$, and MA positions $T^{\left(0\right)}$. Set AO iteration counter l=0.  2:**repeat**  3:   **Step 1: Active Beamforming Optimization**  4:   With fixed $\left\{v_{t}^{\left(l\right)},v_{r}^{\left(l\right)}\right\}$ and $T^{\left(l\right)}$, solve the subproblem (12) for active beamforming.  5:   Obtain updated matrices $\left\{R_{k}^{\left(l+1\right)},R_{s}^{\left(l+1\right)}\right\}$ by using the SCA-based method summarized in Algorithm 1.  6:   **Step 2: Passive Beamforming Optimization**  7:   With fixed $\left\{R_{k}^{\left(l+1\right)},R_{s}^{\left(l+1\right)}\right\}$ and $T^{\left(l\right)}$, solve the subproblem in Section 3.2 for passive beamforming.  8:   Obtain updated vectors $\left\{v_{t}^{\left(l+1\right)},v_{r}^{\left(l+1\right)}\right\}$ by using the SCA-based method summarized in Algorithm 2.  9:   **Step 3: MA Position Optimization**10:   With fixed $\left\{R_{k}^{\left(l+1\right)},R_{s}^{\left(l+1\right)}\right\}$ and $\left\{v_{t}^{\left(l+1\right)},v_{r}^{\left(l+1\right)}\right\}$, solve the subproblem (23) for MA positions.11:   Update the position of each MA sequentially by applying the BCD and SCA techniques to solve the convex subproblem (28), which yields the updated positions $T^{\left(l+1\right)}$.12:   Update the iteration counter: l←l+1.13:**until** the fractional increase of the minimum secrecy rate is below a threshold or the maximum number of iterations is reached.14:*Output:* The optimized active beamforming matrices $\left\{R_{k}^{*},R_{s}^{*}\right\}$, passive beamforming vectors $\left\{v_{t}^{*},v_{r}^{*}\right\}$, and MA positions $T^{*}$.

### 3.4. Computational Complexity Analysis

We now analyze the computational complexity of the proposed AO-based algorithm (Algorithm 4), which is determined by the complexities of the three subproblems solved in each AO iteration. Let $I_{\mathrm{AO}}$ denote the number of iterations for the main AO loop. Let $I_{\mathrm{SCA}1}$ and $I_{\mathrm{SCA}2}$ denote the number of iterations required for the SCA-based Algorithm 1 and Algorithm 2, respectively. Finally, let $I_{\mathrm{BCD}}$ and $I_{\mathrm{SCA}\_m}$ denote the number of iterations for the outer BCD loop and the inner SCA loop (for each antenna *m*) in Algorithm 3, respectively.

- **Algorithm 1 (Active Beamforming Optimization):** This algorithm iteratively solves a Semidefinite Program (SDP), as formulated in (16). The problem involves K+1 positive semidefinite matrix variables of size M×M, along with O(K) linear constraints. The complexity of solving this SDP in each SCA iteration using interior-point methods (IPMs) can be approximated as $O\left({\left(K+1\right)}^{3.5}M^{3.5}\right)$. Therefore, the total complexity of Algorithm 1 is $O\left(I_{\mathrm{SCA}1}\cdot {\left(K+1\right)}^{3.5}M^{3.5}\right)$.
- **Algorithm 2 (Passive Beamforming Optimization):** This algorithm, detailed in Section 3.2, also solves an SDP. The problem involves two main matrix variables ($V_{r},V_{t}$) of size N×N. The complexity per SCA iteration is dominated by solving this SDP, which is $O\left(N^{3.5}\right)$. Thus, the total complexity of Algorithm 2 is $O\left(I_{\mathrm{SCA}2}\cdot N^{3.5}\right)$.
- **Algorithm 3 (MA Position Optimization):** This algorithm uses a BCD approach, iterating *M* times (once for each antenna) within its $I_{\mathrm{BCD}}$ loops. In each of the *M* blocks, it solves a convex subproblem (28) using an inner SCA loop that runs for $I_{\mathrm{SCA}\_m}$ iterations. This subproblem (28) is a small convex program (CP), likely a Second-Order Cone Program (SOCP), with only three scalar variables ($t_{m}\in R^{2}$ and τ) and O(K+M) constraints. Let $C_{\mathrm{CP}}\left(K,M\right)$ denote the low polynomial cost of solving this small CP. The total complexity of Algorithm 3 is $O\left(I_{\mathrm{BCD}}\cdot M\cdot I_{\mathrm{SCA}\_m}\cdot C_{\mathrm{CP}}\left(K,M\right)\right)$.

The overall computational complexity of the proposed Algorithm 4 is the sum of these components within the main AO loop:

(29) $$O\left(I_{\mathrm{AO}}\cdot \left(I_{\mathrm{SCA}1}{\left(K+1\right)}^{3.5}M^{3.5}+I_{\mathrm{SCA}2}N^{3.5}+I_{\mathrm{BCD}}\cdot M\cdot I_{\mathrm{SCA}\_m}\cdot C_{\mathrm{CP}}\left(K,M\right)\right)\right).$$

In typical massive RIS-assisted systems, the number of RIS elements *N* is very large (i.e., N≫M and N≫K). Consequently, the overall complexity is expected to be dominated by the passive beamforming optimization (Algorithm 2), i.e., $O\left(I_{\mathrm{AO}}\cdot I_{\mathrm{SCA}2}\cdot N^{3.5}\right)$.

## 4. Numerical Results

In this section, we provide numerical results to validate the performance of our proposed algorithm in a multi-user, multi-eavesdropper scenario. We consider a three-dimensional Cartesian coordinate system in which the Base Station (BS), equipped with movable antennas, is located at (0,0,2) m. The STAR-RIS, comprising N=64 elements, is deployed at (0,20,2) m. To rigorously test the system’s capabilities, we model a scenario with a total of K=2 legitimate users. One user is located in the transmission space, and the other is located in the reflection space. We also consider two eavesdroppers. One in the transmission space at (−4,20,0) m. And the other one in the reflection space at (4,20,0) m. Meanwhile, the positions of the two users are randomly generated in each Monte Carlo simulation. The user located in the transmission space is uniformly distributed within a circular area centered at (−2,19,0) m. The user located in the reflection space is uniformly distributed within a circular area centered at (2,19,0) m. The optimization problem is to maximize the minimum secrecy rate between the two users.

The channel model for the BS-RIS link is assumed to follow Rician fading with a Rician factor of K=10 dB, reflecting a dominant line-of-sight (LoS) path. All other links are modeled using Rayleigh fading. The large-scale path loss is modeled as $\beta_{0}d^{-\alpha }$, where $\beta_{0}=-30$ dB is the path loss at a reference distance of 1 m. The path loss exponents for the BS-RIS and RIS-Users/Eve links are set to $\alpha_{br}=2.2$ and $\alpha_{ru}=2.8$, respectively. The maximum transmit power at the BS is $P_{\max}=42$ dBm, and the noise power is $\sigma^{2}=-70$ dBm. The carrier frequency is $f_{c}=2.4$ GHz. Our iterative algorithm terminates when the fractional increase of the objective function is below a tolerance of $\epsilon =10^{-4}$ or after a maximum of 15 iterations. The final results are averaged over 200 Monte Carlo trials.

### 4.1. Benchmark Schemes for Comparison

To validate the contributions of the key components in our proposed design, namely the joint optimization of movable antennas and the utilization of STAR-RIS, we consider the following three benchmark schemes:

- **Scheme 1: Reflecting-only RIS**. This scheme replaces the STAR-RIS with a conventional reflecting-only RIS. This scheme quantifies the performance gain by the STAR-RIS.
- **Scheme 2: FPA (Fixed-Position Antenna)**. This scheme replaces the MAs at the BS with a conventional fixed-position uniform linear array (ULA). The positions of the antennas are not subject to optimization. This highlights the performance gain by the optimization of the positions of the MAs.
- **Scheme 3: RPA (Random-Position Antenna)**. The antenna positions are randomly selected in each simulation. This benchmark demonstrates the performance gains from the optimization of the MAs.

### 4.2. Convergence Analysis

**Figure 2:**
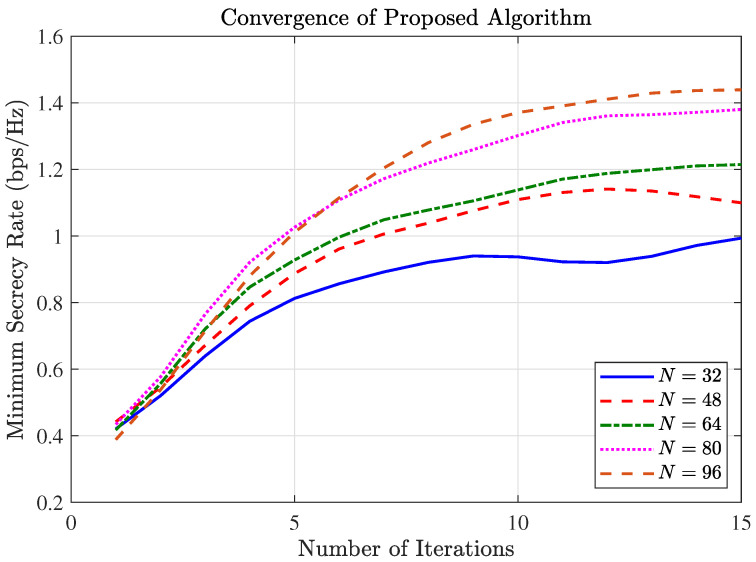
Convergence behavior.

Figure 2 demonstrated the convergence behavior of the proposed AO algorithm. As observed in Figure 2, the secrecy rate increases monotonically and typically reaches a stable plateau after approximately 10 iterations. This behavior confirms that the algorithm satisfies the convergence criterion (fractional increase below $10^{-4}$) well within the maximum limit of 15 iterations. This rapid convergence validates the effectiveness and computational efficiency of the proposed algorithm. The convergence behavior confirms that the SCA and SDR techniques are effective at finding high-quality solutions.

### 4.3. Impact of BS Transmit Power

**Figure 3:**
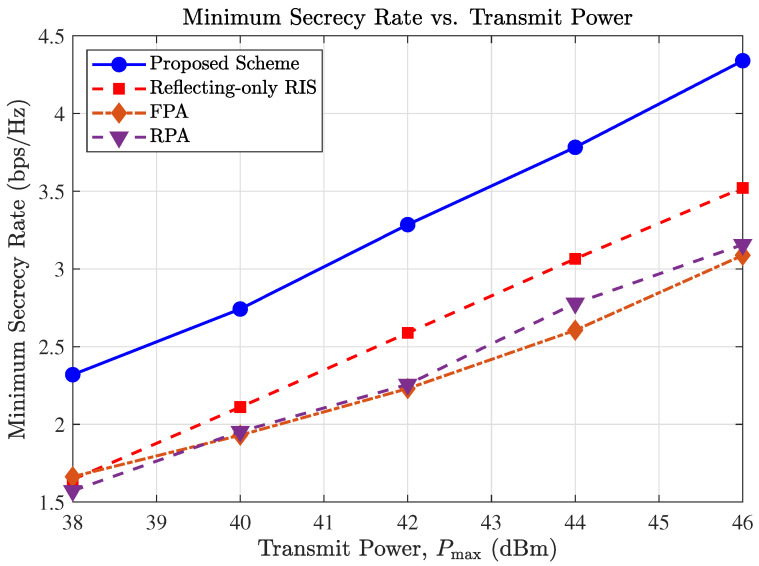
Secrecy rate versus the total transmit power at the BS ($P_{\max}$).

Figure 3 illustrates the minimum secrecy rate versus the maximum transmit power at the BS, $P_{\max}$. As expected, the secrecy rates of all schemes improve as the transmit power increases. However, the proposed MA-aided STAR-RIS scheme exhibits the steepest slope, indicating superior power efficiency compared to the benchmarks.

Specifically, the performance gap between the proposed scheme and the fixed-position antenna (FPA) benchmark widens significantly in the high-SNR regime. This trend can be attributed to the spatial flexibility of MAs. In high-power regimes, the system is often limited by interference or channel correlation rather than noise. Fixed antennas (FPA) may be stuck in positions with deep fading or high correlation between legitimate and eavesdropping channels. In contrast, MAs can dynamically maneuver to spatial “sweet spots” that maximize the channel gain difference between the legitimate user and the eavesdropper. This capability allows the system to convert increased transmit power into secrecy capacity more effectively.

Furthermore, the proposed scheme significantly outperforms the “Reflecting-only RIS” benchmark. This confirms the critical advantage of the STAR-RIS architecture, which enables full-space coverage ($K_{t}\cup K_{r}$) to serve users in both transmission and reflection regions simultaneously, whereas a conventional reflecting-only RIS fails to effectively cover the transmission space.

### 4.4. Impact of the Number of RIS Elements

**Figure 4:**
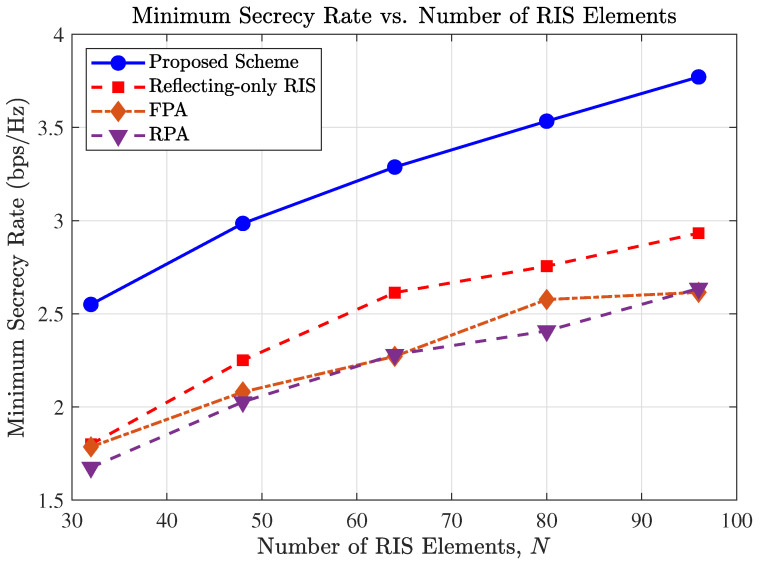
Secrecy rate versus the number of STAR-RIS elements (*N*).

Figure 4 depicts the impact of the number of STAR-RIS elements, *N*, on the minimum secrecy rate. It is observed that increasing *N* leads to substantial performance gains across all RIS-assisted schemes, primarily due to the enhanced passive beamforming gain and higher spatial resolution provided by the larger aperture.

Notably, the proposed scheme maintains a distinct and growing advantage over the FPA scheme as *N* increases. This highlights a unique synergistic effect between the MAs and the STAR-RIS. The optimization of MA positions does not merely improve the direct links; crucially, it reconfigures the BS-to-RIS channel ($H_{br}$) to align with the STAR-RIS array response. By moving the antennas, the BS can “illuminate” the STAR-RIS more effectively, maximizing the signal power that reaches the RIS elements.

Consequently, the large-scale STAR-RIS can manipulate a stronger incident signal, leading to a multiplicative gain in the cascaded link. In contrast, the FPA scheme is constrained by a fixed, potentially suboptimal $H_{br}$, which creates a bottleneck that prevents the system from fully exploiting the potential of a large *N*. The “Reflecting-only RIS” and “RPA” schemes exhibit the lowest performance, further validating the necessity of the proposed joint design.

## 5. Conclusions

In this paper, we propose a novel secure integrated sensing and communication system enhanced by movable antennas and a STAR-RIS. To address security threats from multiple eavesdroppers, we investigated the joint optimization of active beamforming, passive beamforming, and MA positions to maximize the minimum secrecy rate among all legitimate users. We designed an efficient iterative algorithm based on the Alternating Optimization framework to solve the formulated non-convex problem. Specifically, Semidefinite Relaxation (SDR) and Successive Convex Approximation (SCA) techniques were leveraged for the beamforming subproblems, while a BCD-SCA-based method was developed for the challenging MA position optimization. Numerical results validated the superior performance and rapid convergence of the proposed algorithm, demonstrating significant secrecy rate gains over various benchmark schemes. Future work will extend this framework to scenarios with imperfect channel state information (CSI) and explore deep reinforcement learning-based solutions.

## Figures and Tables

**Table 1:** Description of key symbols.

Symbol	Description
System Parameters	
*M*	Number of movable antennas (MAs) at the BS
*N*	Number of STAR-RIS elements
*K*	Number of legitimate users
*J*	Number of eavesdroppers
$P_{\max}$	Maximum transmit power at the BS
$P_{\mathrm{th}}$	Minimum received power requirement for sensing
$\sigma_{k}^{2},\sigma_{j}^{2},\sigma_{s}^{2}$	Noise power at the user *k*, eavesdropper *j*, and sensing receiver
Channel Matrices and Vectors	
$H_{\mathrm{br},k}\in C^{1\times M}$	Channel vector from the BS to user *k*
$H_{\mathrm{be},j}\in C^{1\times M}$	Channel vector from the BS to eavesdropper *j*
$H_{\mathrm{sr}}\in C^{N\times M}$	Channel matrix from the BS to the STAR-RIS
$G_{\mathrm{sr},k}\in C^{1\times N}$	Channel vector from the STAR-RIS to user *k*
$G_{\mathrm{se},j}\in C^{1\times N}$	Channel vector from the STAR-RIS to eavesdropper *j*
$h_{\mathrm{ss}}\in C^{M\times 1}$	Echo channel vector from the sensing target to the BS
Optimization Variables	
$W\in C^{M\times M}$	Active beamforming matrix at the BS
$w_{k}\in C^{M\times 1}$	Beamforming vector for user *k*
$w_{s}\in C^{M\times 1}$	Dedicated sensing beamforming vector
$p_{m}\in R^{2\times 1}$	Position coordinates of the *m*-th MA
Θ	STAR-RIS coefficient matrix, defined as $\mathrm{diag}(\beta e^{j\theta })$
β	Amplitude coefficients of the STAR-RIS, i.e., ${\left[\sqrt{\beta_{1}},\ldots ,\sqrt{\beta_{N}}\right]}^{T}$
θ	Phase shifts of the STAR-RIS, i.e., ${\left[\theta_{1},\ldots ,\theta_{N}\right]}^{T}$
$R_{\sec}$	Minimum secrecy rate among all legitimate users
Other Variables	
$\gamma_{k},\gamma_{j}$	Signal-to-interference-plus-noise ratio (SINR) at user *k* and eavesdropper *j*
$C_{k},C_{j}$	Channel capacity of user *k* and eavesdropper *j*

## Data Availability

The original contributions presented in this study are included in the article. Further inquiries can be directed to the corresponding author.

## References

[1] Guo S., Zheng H., Zhang Y., Yan J. Joint Secure Transmit Beamforming Designs for Physical Layer Security in MIMO-ISAC System. Proceedings of the 2024 IEEE International Conference on Signal, Information and Data Processing (ICSIDP).

[2] Zhang L., Wang Y., Chen H., Cao Y. (2025). Physical-Layer Security of the NOMA-Assisted ISAC Systems Under Near-Field Scenario. IEEE Internet Things J..

[3] Zhao B., Qiu T., Ren G., Jin Z., Liu Z. (2025). RSMA-Enhanced Physical Layer Security for ISAC Systems. IEEE Wirel. Commun. Lett..

[4] Xiu Y., Lyu W., Yeoh P., Ai Y., Li Y., Wei N. (2025). Improving Physical-Layer Security in ISAC-AAV System: Beamforming and Trajectory Optimization. IEEE Trans. Veh. Technol..

[5] Jia H., Li X., Ma L. (2024). Physical Layer Security Optimization With Cramér–Rao Bound Metric in ISAC Systems Under Sensing-Specific Imperfect CSI Model. IEEE Trans. Veh. Technol..

[6] Xing Z., Wang R., Yuan X. (2024). Reconfigurable Intelligent Surface Aided Physical-Layer Security Enhancement in Integrated Sensing and Communication Systems. IEEE Trans. Veh. Technol..

[7] Mu X., Liu Y., Guo L., Lin J., Schober R. (2022). Simultaneously Transmitting and Reflecting (STAR) RIS Aided Wireless Communications. IEEE Trans. Wirel. Commun..

[8] Liu Z., Li X., Ji H., Zhang H. Exploiting STAR-RIS for Physical Layer Security in Integrated Sensing and Communication Networks. Proceedings of the 2023 IEEE 34th Annual International Symposium on Personal, Indoor and Mobile Radio Communications (PIMRC).

[9] Wang C., Wang C.C., Li Z., Ng D.W.K., Wong K.K. PHY Security Enhancement Exploiting STAR-RIS for Dual-Functional Radar-Communication. Proceedings of the 2023 IEEE International Conference on Communications Workshops (ICC Workshops).

[10] Xie Z., Liu Y., Yi W., Wu X., Nallanathan A. (2024). Physical Layer Security for STAR-RIS-NOMA: A Stochastic Geometry Approach. IEEE Trans. Wirel. Commun..

[11] Pala S., Singh K., Taghizadeh O., Pan C., Dobre O.A., Duong T.Q. (2025). Robust and Secure Multi-User STAR-RIS-Aided Communications: Optimization Versus Machine Learning. IEEE Trans. Commun..

[12] Xiao H., Hu X., Li A., Wang W., Yang K. (2025). Robust Full-Space Physical Layer Security for STAR-RIS-Aided Wireless Networks: Eavesdropper With Uncertain Location and Channel. IEEE Trans. Wirel. Commun..

[13] Guo L., Jia J., Mu X., Liu Y., Chen J., Wang X. (2025). Joint Secure and Covert Communications for Active STAR-RIS Assisted ISAC Systems. IEEE Trans. Wirel. Commun..

[14] Xiao H., Hu X., Li A., Wang W., Su Z., Wong K.K., Yang K. STAR-RIS-Assisted Joint Physical Layer Security and Covert Communications. Proceedings of the 2023 IEEE 98th Vehicular Technology Conference (VTC2023-Fall).

[15] Xiao H., Hu X., Li A., Wang W., Su Z., Wong K.K., Yang K. (2024). STAR-RIS Enhanced Joint Physical Layer Security and Covert Communications for Multi-Antenna mmWave Systems. IEEE Trans. Wirel. Commun..

[16] Ye R., Peng Y., Yue M., Lee J. (2025). Physical Layer Security for IoT Application With Assistance of Active STAR-RIS. IEEE Internet Things J..

[17] Zhu L., Ma W., Zhang R. (2024). Modeling and Performance Analysis for Movable Antenna Enabled Wireless Communications. IEEE Trans. Wirel. Commun..

[18] Lyu W., Yang S., Xiu Y., Zhang Z., Assi C., Yuen C. Flexible Beamforming for Movable Antenna-Enabled Integrated Sensing and Communication. Proceedings of the 2024 IEEE 24th International Conference on Communication Technology (ICCT).

[19] Zou J., Xu H., Wang C., Xu L., Sun S., Meng K., Masouros C., Wong K.-K. (2024). Shifting the ISAC Trade-Off With Fluid Antenna Systems. IEEE Wirel. Commun. Lett..

[20] Xiang W., Chen Y., Zhang X., Lu Z., Wen X. Joint antenna position and transmit signal optimization for ISAC system with movable antenna array. Proceedings of the 2024 IEEE 35th International Symposium on Personal, Indoor and Mobile Radio Communications (PIMRC).

[21] Xiu Y., Yang S., Lyu W., Yeoh P.L., Li Y., Ai Y. (2025). Movable Antenna Enabled ISAC Beamforming Design for Low-Altitude Airborne Vehicles. IEEE Wirel. Commun. Lett..

[22] Peng S., Zhang C., Xu Y., Wu Q., Zhu L., Ou X., He D. Joint Antenna Position and Beamforming Optimization with Self-Interference Mitigation in Movable Antenna Aided ISAC System. Proceedings of the 2025 IEEE Wireless Communications and Networking Conference (WCNC).

[23] Ding J., Zhou Z., Shao X., Jiao B., Zhang R. (2025). Movable Antenna-Aided Near-Field Integrated Sensing and Communication. IEEE Trans. Wirel. Commun..

[24] Chen L., Zhao M.M., Zhao M.J., Zhang R. (2025). Antenna Position and Beamforming Optimization for Movable Antenna Enabled ISAC: Optimal Solutions and Efficient Algorithms. IEEE Trans. Signal Process..

[25] Wu H., Ren H., Pan C., Zhang Y. (2025). Movable Antenna-Enabled RIS-Aided Integrated Sensing and Communication. IEEE Trans. Cogn. Commun. Netw..

[26] Xiong W., Zhong K., Xiao Z., Lin J., Li Q. Secure Analog Beamforming Design for Wireless Communication Systems With Movable Antennas. Proceedings of the ICASSP 2025-2025 IEEE International Conference on Acoustics, Speech and Signal Processing (ICASSP).

[27] Cheng Z., Ouyang C., Zhang X. Movable Antenna Aided Physical Layer Security with No Eavesdropper CSI. Proceedings of the ICASSP 2025-2025 IEEE International Conference on Acoustics, Speech and Signal Processing (ICASSP).

[28] Liu P., Si J., Cheng Z., Li Z., Hu H. (2025). Movable-Antenna Enabled Covert Communication. IEEE Wirel. Commun. Lett..

[29] Dong X., Lyu W., Yang R., Xiu Y., Mei W., Zhang Z. (2025). Movable Antenna Enhanced Secure Simultaneous Wireless Information and Power Transfer. IEEE Commun. Lett..

[30] Hung H.L., Huy N.H., Luong N.C., Pham Q.V., Niyato D., Hoa N.T. (2025). Beamforming Design for Physical Security in Movable Antenna-aided ISAC Systems: A Reinforcement Learning Approach. IEEE Trans. Veh. Technol..

